# Current Status of Legislation on Dietary Products for Sportspeople in a European Framework

**DOI:** 10.3390/nu9111225

**Published:** 2017-11-08

**Authors:** José Miguel Martínez-Sanz, Isabel Sospedra, Eduard Baladía, Laura Arranz, Rocío Ortiz-Moncada, Angel Gil-Izquierdo

**Affiliations:** 1 Nursing Department, Faculty of Health Sciences, University of Alicante, 03690 Alicante, Spain; isospedra@ua.es; 2 Research Group on Food and Nutrition (ALINUT), University of Alicante, 03690 Alicante, Spain; e.baladia@academianutricion.org (E.B.); rocio.ortiz@ua.es (R.O.-M.); angelgil@cebas.csic.es (A.G.-I.); 3 Evidence-Based Nutrition Network (RED-NuBE), Spanish Academy of Nutrition and Dietetics (AEND), 31006 Navarra, Spain; 4 Department of Nutrition and Food Science, Faculty of Pharmacy, University of Barcelona, 08007 Barcelona, Spain; gana_bcn@yahoo.es; 5 Department of Community Nursing, Preventive Medicine and Public Health and History of Science Health, University of Alicante, 03690 Alicante, Spain; 6 Quality, Safety, and Bioactivity of Plant Foods Group, Department of Food Science and Technology, CEBAS-CSIC, University of Murcia, 30100 Murcia, Spain

**Keywords:** food legislation, health claims, nutritional ergogenic aids

## Abstract

The consumption of nutritional ergogenic aids is conditioned by laws/regulations, but standards/regulations vary between countries. The aim of this review is to explore legislative documents that regulate the use of nutritional ergogenic aids intended for sportspeople in a Spanish/European framework. A narrative review has been developed from official websites of Spanish (Spanish Agency of the Consumer, Food Safety, and Nutrition) and European (European Commission and European Food Safety Authority) bodies. A descriptive analysis of documents was performed. Eighteen legislative documents have been compiled in three sections: (1) Advertising of any type of food and/or product; (2) Composition, labeling, and advertising of foods; (3) Nutritional ergogenic aids. In spite of the existence of these legal documents, the regulation lacks guidance on the use/application of nutritional ergogenic aids for sportspeople. It is essential to prevent the introduction or dissemination of false, ambiguous, or inexact information and contents that induce an error in the receivers of the information. In this field, it is worth highlighting the roles of the European Food Safety Authority and the World Anti-Doping Agency, which provide information about consumer guidelines, prescribing practices, and recommendations for the prudent use of nutritional ergogenic aids.

## 1. Introduction

Ergogenic aids have been defined as substances or methods used to improve endurance, total fitness level, and sports performance; they could be anything that gives a mental or physical edge while exercising or competing. These aids are classified as nutritional, pharmacologic, physiologic, or psychological [1,2]. In the field of nutrition, foods and food components that can improve the capacity of an individual to perform an exercise task have also been described as ergogenic aids [3].

In the context of sport, nutritional ergogenic aids are commonly known as dietary or sport supplements, sports food, or food aids, and have been used for different purposes: in particular, to increase energy, maintain strength, health and the immune system, enhance performance, and prevent nutritional deficiencies [4,5,6,7]. However, the use of sports food involves both benefits and risks. Its inadequate consumption can cause adverse effects on the athlete’s health [6,8]. An example of these undesirable effects are unintentional doping situations, caused by the intake of nutritional ergogenic aids containing substances prohibited by the World Anti-Doping Agency (WADA) [9].

The consumption of nutritional ergogenic aids by athletes is conditioned by specific laws, regulations, or instructions. Such legislation should provide advice or recommendations regarding the usage, dose, security, and any precautions and warnings for these substances. It should also provide information about their market access and availability, as well as their efficiency with respect to enhancing sport performance. 

These are general principles of action on public health to ensure that the population can achieve or maintain the highest standard of health [10]. However, some popular products are marketed as ergogenic aids despite a lack of objective evidence to support claims of an ergogenic effect [11].

Standards and regulations on nutritional ergogenic aids vary between countries and also between different types of products. In the European Union and its member states, several provisions on sport foods can be found. All of them include labeling (health or performance claims in labels), safety, and marketing aspects and the contents of vitamins, minerals, and other substances [12,13]. However, currently there is no specific legislation on nutritional ergogenic aids.

Misleading advertising as well as an incomplete labeling may also have consequences for the health of the consumers due to a chemical risk linked to doping substances, if non-approved ingredients are used or contamination occurs [14,15].

Beyond European borders, similar situations can be found. In the United States, the Food and Drug Administration (FDA), broadly speaking, regulates quality and the Federal Trade Commission supervises the marketing and advertising of dietary supplements [16]. According to the Dietary Supplement Health and Education Act (DSHEA) [17], dietary supplements, including nutritional ergogenic aids, that are not intended to diagnose, treat, cure, or prevent any disease currently do not need to be evaluated by the FDA prior to their commercialization. The manufacturers are responsible for the determination of the purity, benefits, efficacy, safety, and compositional specifications that the supplement is required to meet [18].

On the basis of the level of scientific evidence and its practical applications, the American Dietetic Association and the Australian Institute of Sport proposed a provisional classification of nutritional ergogenic aids: (1) Supported for use in specific situations in sport using evidence-based protocols; (2) Deserving of further research and could be considered for provision to athletes under a research protocol or case-managed monitoring situation; (3) Having little meaningful proof of beneficial effects; (4) Banned or at high risk of contamination with substances that could lead to a positive drug test [19].

The absence of supporting legislation in Europe contributes to the misleading advertising. As a result of this lack of legislation on nutritional ergogenic aids, the companies can make unsubstantiated claims about the efficacy of the products. Advertisements and health/performance claims in labels with references to improved athletic performance without any scientific evidence can be found on the market.

Thus, since there is no specific legislation on nutritional ergogenic aids, the aim of the present study is to explore legislative documents that regulate the use of nutritional ergogenic aids intended for sportspeople in the Spanish and European frameworks.

## 2. Materials and Methods

The methodology was developed by different strategies, as follows:

(1) A narrative review of legislative documents related to nutritional ergogenic aids, especially for sportspeople, has been developed. The information was extracted from the official websites of Spanish and European bodies. A legislative document is considered to be the laws, regulations, and/or standards laid down by the competent authority in the national or European field.

The search for Spanish documents was carried out on the website of the Spanish Agency for the Consumer, Food Safety, and Nutrition (AECOSAN) of the Spanish Ministry of Health, Social Policy, and Equality and, in particular, on the “legislation” section (http://aesan.msssi.gob.es/AESAN/web/legislacion/seccion/especifica_ambito_alimentario.shtml). The “General” category was consulted to obtain information about “Labeling and advertising of foods” and the “By Sectors” category was explored to compile information about “Food products for special groups”.

In the search for European legislation, the section “Dietetic Foods/Foods for specific groups” on the European Commission website was consulted: http://ec.europa.eu/food/safety/labelling_nutrition/special_groups_food/sportspeople/index_en.htm. Also consulted was the section “The Panel on Dietetic Products, Nutrition and Allergies (NDA)” from the European Food Safety Authority (EFSA) (http://www.efsa.europa.eu/en/panels/nda).

(2) The selection process was performed by two independent reviewers, reaching a consensus and finally approving its inclusion or exclusion.

(3) The selection of the compiled documents was carried out using classification criteria according to the information contained in the publication. Three sections were established:

- Section 1: legislative documents on advertising (for every type of products including foods).
- Section 2: legislative documents on the composition, labeling, and advertising of foods (only for foods).
- Section 3: specific legislative documents on nutritional ergogenic aids (only nutritional/dietary supplements).

## 3. Results

A total of 18 relevant legislative documents have been compiled. All of them deal with nutritional ergogenic aids in a general or specific way and in the Spanish or European framework. For the sake of clarity, the documents are grouped in sections as follows: 

Section 1, documents that refer to the advertising for any type of food and/or product. Six documents applicable in Spain were found; the results are shown in Table 1. 

Section 2, laws and legislative records related to the composition, labeling, and advertising that affect all food and products. Six documents, mostly applicable in Europe, were compiled (Table 2).

Section 3, this section contains the main specific legislative documents about foodstuffs intended for particular nutritional uses. Among them are the foods intended to satisfy the expenditure of intense muscular effort, especially for sportspeople. This section also includes reports issued by the EFSA on the health claims made for nutritional ergogenic aids. Table 3 shows the information about the six documents found.

## 4. Discussion

In the present work the importance of the creation of the EFSA in 2002 is emphasized. This agency is an essential asset for Europe’s food security in the context of public health. 

In the European framework, and according to Action plan No. 55 of the European Commission, there are several legislative documents for food destined to alleviate intense muscle loss, particularly in athletes. Nevertheless, despite the existence of the EFSA, the analyses of legislative documents uncovered ambiguity in the regulations.

The documents related to laws and resolutions in Spain and Europe are orientated towards the general regulation of advertising and food marketing. However, specific documents referring to nutritional ergogenic aids show more ambiguity or are inconsistent or are inexistent. 

Action plan No. 56 of the European Commission (2001) has been reflected in Directive 2009/39/C [33] and in Regulation (EC) No. 953/2009 [34]. Both documents highlight the need to create a more specific legislation for ergonutritional aids. However, in both of them, ergonutritional aids and sport foods are considered together as the group of “foods intended to meet the expenditure of intense muscular effort, especially for sportsmen”.

The guidance of the EFSA on the scientific requirements for health claims related to physical performance [36] represents the views of the NDA panel based on its experience with the evaluation of health claims for physical performance, endurance capacity, muscle function, and physiological effects. This is a compilation about scientific opinions from NDA panel that have been approved by the European Commission to be applied on the European Union countries.

### 4.1. Legislative Documents in Spain

This guide is based on Regulation (EC) No. 1924/2006 [28] and its subsequent amendments, concerning nutrition and health claims made for foods, and also on Regulation (EU) No. 1169/2011 [30], concerning the provision of food information to consumers. 

Domestically, in Spain, several regulations on the advertising of any food and/or product exist. These establish common considerations related to unfair competition and illicit advertising on labeling [20,21,23,24]. 

In this context, it is worth noting the agreement signed in 1998 between the Ministry of Health and Consumer Affairs and the Spanish Federation of Food and Drink Industries (FIAB). This provides criteria for assessing the conformity of a product with the general safety requirements and the right of consumers to obtain accurate, truthful, and reliable information. These criteria have subsequently been reinforced by Law 17/2011, of 5 July 2011, on food safety and nutrition [38] and more especially by the Co-regulation Codex on food and beverages advertising targeted at minors, obesity prevention, and health (PAOS Codex) [39].

In Spain, sports food has been regulated for 40 years through the Royal Decree 2685/1976 “Healthcare Regulation on Formula Foods for Use in Weight Control Diets and/or in Special Situations” [32]. This Royal Decree defined the “food destined to those people who make extraordinary efforts or live in special environmental conditions” as “food that provides complementary nutrients” in Section 3.1.2.2 and this is also included in Section 3.1.2 as “complementary food for situations of great physical exertion”. Even so, the Annex constitutes an indication of the need to create specific regulations for sports food.

### 4.2. Legislative Documents in Europe

In February 2001, the Health & Consumer Protection Directorate-General of the European Commission ordered the Scientific Committee on Food (SCF) (European Commission. Health and Consumer Protection, 2001) to write a report on the food composition and specification of food intended to meet the expenditure of intense muscular effort, especially for sportsmen [40]. The document concluded that a well-balanced diet is the basic nutritional requirement for athletes. Nevertheless, taking into consideration the distinct aspects of intense muscular exercise—such as intensity, duration, and frequency as well as specific constraints like time and convenience—individuals can benefit from particular foods or food ingredients. Specially adapted nutritious foods or fluids may help to solve specific problems so that an optimal nutritional balance can be reached. On the basis of such considerations, four food categories, for which essential requirements were formulated, were identified: (1) Carbohydrate-rich energy food products; (2) Carbohydrate–electrolyte solutions; (3) Protein and protein components; (4) Supplements and other food components. 

This report was the first step in the categorization and legislation of sport foods, as indicated in the Action plan No. 55 of the White Paper on Food Safety [26].

Directive 2009/39/EC states the conditions that foodstuffs must comply with in order to bear the words dietetic or dietary. Foodstuffs for particular nutritional uses are defined as “foodstuffs which, owing to their special composition or manufacturing process, are clearly distinguishable from foodstuffs for normal consumption, which are suitable for their claimed nutritional purposes and which are marketed in such a way as to indicate such suitability”. This Directive also establishes that “A particular nutritional use shall fulfil the particular nutritional requirements of certain categories of persons who are in a special physiological condition and who are therefore able to obtain special benefit from controlled consumption of certain substances in foodstuffs”. Among the products destined for a special diet, adapted food for intense muscle wastage is found, especially for athletes [33]. Consequently, from the evaluation of new substances by the EFSA, the necessity to update and complete lists of substances that can be added to dietetic products through Regulation (EC) No. 953/2009 arises [34]. Substances like vitamins, minerals, amino acids, l-carnitine, taurine, nucleotides, choline, and inositol are on this list. These compounds are frequently used for the preparation of dietetic products and sport foods, but their use to improve sport performance has questionable benefits [41,42].

In addition to the lack of scientific evidence about sport performance benefits, important inaccuracies and legal loopholes can be found in this Regulation. Two examples are the sentence “the inclusion of substances in the list of those that may be used in the manufacture of foodstuffs for particular nutritional uses does not mean that their addition to those foodstuffs is necessary or desirable” and point 2 of Article 2: “also substances not belonging to the categories appearing in the Annex to this Regulation may be added for specific nutritional purposes in the manufacture of foods for particular nutritional uses”.

Directive 2009/39/CE foresees a specific legislation of foods intended to meet the expenditure of intense muscular effort, especially for sportsmen [33].

Due to these legal loopholes, the European Commission has elaborated recently the Regulation (EU) No. 609/2013 on food intended for infants and young children, food for special medical purposes, and total diet replacement for weight control and has repealed Council Directive 92/52/EEC [35]. This suppose a clear fault on food safety and consequently, inefficient control of these nutritional/dietary supplements. This new Regulation will replace Directive 2009/39/CE [19] and specifies that “food intended to meet the expenditure of intense muscular effort, especially for sportsmen, no successful conclusion could be reached as regards the development of specific provisions due to widely diverging views among the Member States and stakeholders concerning the scope of specific legislation, the number of subcategories of food to be included, the criteria for establishing compositional requirements and the potential impact on innovation in product development. Therefore, specific provisions should not be developed at this stage”. Section 13 of this Regulation discusses food intended for sportspeople and mentions that “By 20 July 2015, the Commission shall, after consulting the Authority, present to the European Parliament and to the Council a report on the necessity, if any, of provisions for food intended for sportspeople. Such a report may, if necessary, be accompanied by an appropriate legislative proposal”. Considerations previous to this Regulation indicate that the report of 28 February 2001 of the SCF should be taken into account [40]. Today, there is no law/rule on these products and this type of food will be exclusively governed by horizontal rules of food law.

### 4.3. Specific Legislation on Nutritional Ergogenic Aids or Sports Food

Until now, the European Commission has not proposed any specific legislation concerning this field, but the Directorate General for Health and Food Safety ordered that a report be drawn up on food for sportspeople [35]. This report indicates that there is no universally accepted definition of what constitutes “sports food”. The scope of the report is not limited only to foods available on the market under Directive 2009/39/EC, but considers all products specifically targeting sportspeople regardless of their method of market placement. 

Its objective is to provide the information missing in current legislation and aims to provide: (1) A description and analysis of the market of foods intended for sportspeople; (2) A description and analysis of the different marketing techniques and practices used for foods intended for sportspeople, with particular attention to the use of nutrition and health claims; (3) A description and analysis of the consumers of foods intended for sportspeople, with particular focus on their behavior, interest, and understanding; (4) A description of the national legislation in the 28 Member States related to foods intended for sportspeople, when this exists, and an analysis of how this is performing; and (5) A description and analysis of the legislations in the main trading partner countries related to foods intended for sportspeople. 

The study will help the development of a legislative proposal, in which Regulation No. 609/2013 [35] will be applied, repealing Directive 2009/39/E [33]. Foodstuffs currently considered as “dietary products”, but not included in Regulation 609/2013, will be regulated by legal acts applicable to all foods so long as they do not contradict the horizontal rules of EU food law after 20 July 2016 [37,38]: (a) Regulation (EC) No. 1924/2006 on nutrition and health claims made on foods [28]; (b) Regulation (EC) No. 353/2008, establishing the implementation of rules for applications for authorization of health claims as provided for in Article 15 of Regulation (EC) No. 1924/2006 of the European Parliament and of the Council [29]; (c) Regulation (EC) No. 1925/2006 on the addition of vitamins and minerals and certain other substances to foods [43]; (d) Regulation (EU) No. 1169/2011 on the provision of food information to consumers [30]; (e) Directive 2002/46/EC on the approximation of the laws of the Member States relating to food supplements [44]; and (f) Regulation (EC) No. 258/97 on novel foods and novel food ingredients [45] (this Regulation will be replaced, as of 1 January 2018, by Regulation (EU) 2015/2283 on novel food [46]). Compared to the legislative framework of Directive 2009/39/EC, there are differences regarding how and which information can be provided to the consumer and regarding the composition of the product concerned. 

Regulation (EC) No. 1924/2006 provides the legal framework and the rules for these statements. It would facilitate the consumer’s choice, while avoiding ambiguous, illegal, misleading, or false advertising [47]. While nutritional declarations are strictly, explicitly, and clearly defined in the regulation, declarations of healthy properties must be requested, examined based on generally accepted scientific tests, and be finally accepted by the European Union in positive lists [48]. 

General documental requirements are established for the use of health claims [49] and also there are more specific requirements for nutritional ergogenic aids [36]. Health claims should adequately and sufficiently demonstrate that they are based on and substantiated by generally accepted scientific evidence, by taking into account the totality of the available scientific data and by weighing the evidence. Health claims must satisfy the following principles [47]: (a) a comprehensive and systematic review of data from human subjects should be conducted; (b) published and unpublished data must be included; (c) positive and negative data must be evaluated; (d) priority must be given (in this order) to intervention studies, observational studies, human studies, animal model studies, and studies with cell models, and (e) the methodological quality of intervention studies, observational studies, and meta-analysis must be evaluated.

These requirements, that would demonstrate a clear cause–effect relationship between nutritional ergogenic aids and the declared effect, could be the cause of the scarce approval of the declarations of healthy properties related to sports performance. While more than 2200 general declarations have been presented, just a few of them have been approved [31]. Some examples of claims for substances approved by the EFSA are creatine (increases physical performance in successive bursts of short-term, high-intensity exercise) [50], carbohydrates (contribute to the recovery of normal muscle function after highly intensive and/or long-lasting physical exercise leading to muscle fatigue and the depletion of glycogen stores in skeletal muscle) [51], carbohydrate–electrolyte solutions (contribute to the maintenance of endurance performance during prolonged endurance exercise or enhance the absorption of water during physical exercise) [52], and vitamin C (contributes to maintaining the normal function of the immune system during and after intense physical exercise) [53]. These approvals are based on scientific opinions issued by the American Academy of Nutrition and Dietetics [41] and the Australian Sport Commission and these substances have a relationship with sportspeople’s health and performance [19]. For example, the health effect of carbohydrate–electrolyte solutions is maintenance of endurance performance, while that of Vitamin C is to maintain the function of the immune system during and after extreme physical exercise. It should be noted that caffeine is an exceptional case. Although it has a positive evaluation by the EFSA [54], it was not authorized by the European Commission. The latest scientific opinion, after public consultation, informed that a dosage of 3 mg/kg (200 mg approx.) consumed within 2 h before intense physical exercise in normal environmental conditions does not show any problem [55].

Nutrition claims and the health claims made for proteins, vitamins, and minerals based on Regulation (EC) No. 1924/2006 are, by and large, general in nature but are also applicable to nutritional ergogenic aids [28,31].

According to Directive 2002/46/EC, a food supplement “means foodstuffs the purpose of which is to supplement the normal diet and which are concentrated sources of nutrients or other substances with a nutritional or physiological effect, alone or in combination, marketed in dose form, namely forms such as capsules, pastilles, tablets, pills and other similar forms, sachets of powder, ampoules of liquids, drop dispensing bottles, and other similar forms of liquids and powders designed to be taken in measured small unit quantities” [44]. These represent the most common forms of presentation for companies commercializing nutritional ergogenic aids. The labeling of nutritional ergogenic aids is regulated by Regulation No. 1169/2011 on the provision of food information to consumers [30]. 

With the entry into force of Regulation No. 1169/2011, nutritional labeling became compulsory. In addition to the declaration of particular elements of the qualitative and quantitative compositions, the declaration of allergens and nano-nutrients has become mandatory, as well as an increase in the size of the letters. Regulation No. 1169/2011 amends rule 7 of Regulation (CE) No. 1924/2006: “Nutrition labeling of products on which a nutrition and/or health claim is made shall be mandatory, with the exception of generic advertising. The information to be provided shall consist of that specified in Article 30(1) of Regulation (EU) No. 1169/2011 of the European Parliament and of the Council of 25 October 2011 on the provision of food information to consumers (*). Where a nutrition and/or health claim is made for a nutrient referred to in Article 30(2) of Regulation (EU) No. 1169/2011 the amount of that nutrient shall be declared in accordance with Articles 31 to 34 of that Regulation” and “The amount(s) of the substance(s) to which a nutrition or health claim relates that does not appear in the nutrition labeling shall be stated in the same field of vision as the nutrition labeling and be expressed in accordance with Articles 31, 32 and 33 of Regulation (EU) No. 1169/2011. The units of measurement used to express the amount of the substance shall be appropriate for the individual substances concerned”.

On 24 September 2015, the EFSA issued a technical report called “Scientific and technical assistance on food intended for sportspeople” based on the SCF report of 2001 [40,37] and on EFSA scientific opinions about sports. The report does not take into account new scientific reports about nutritional ergogenic aids published in recent years, after the issue of its scientific views. Because of this, the report could be outdated and out of context [41,56,57]. On the other hand and according to the Directorate General for Health and Food Safety, the report should contribute to the development of a legislative proposal. However, none of the points it mentions have been accomplished.

The majority of national competent authorities believe that the existing horizontal rules of food law are either quite suitable or very suitable for the regulation of sports food. Six national competent authorities have recognized the need for specific rules for sports food [58].

### 4.4. Considerations of the World Anti-Doping Agency

Finally, the list of forbidden substances issued by the WADA [59] and its World Anti-Doping Code [60] must be heeded. Although this information is important because prohibited substances could be a health risk for athletes and their sporting careers [61], the WADA reports are not taken into account in the current legislation on control measures, product supervision, and food complements that contain substances prohibited in sports activities. The WADA warns that dietetic products and herbal products, especially those destined for sport, can contain non-declared substances that could give positive results in anti-doping controls (ephedrine and anabolic substances). The agency also points out that control policies regarding dietetic products are usually quiet lax [62]. 

The scientific literature contains studies that prove the presence of doping substances in supplements like growth hormones [41], androgen receptor modulators, anabolic hormones [63,64,65], and stimulants [66]. Many supplements contain hazardous substances, such as illegal anabolic steroids, that have serious known side effects. In more than 15% of the supplements analyzed, substances were identified at concentrations that are potential positives in “anti-doping” tests and with potential secondary effects for consumers [67,68,69]. The main causes were cross-contamination during manufacturing, processing, or packaging, poor quality control, or bad labeling [14,70,71]. Studies related to the WADA considerations recommend the establishment of better and more effective controls in the elaboration and commercialization of dietetic products. Hence, the WADA has approved programs that guarantee the quality of nutritional ergogenic aids. The quality of the products, suppliers, factory installations, and anti-doping laboratories is certified with a logo that guarantees that these products do not contain prohibited substances.

## 5. Conclusions

Currently, legislation related to the regulation and application of nutritional ergogenic aids or sports food products can be found in the following documents: Regulation (EU) No. 1169/2011, Regulation (EC) No. 353/2008, Regulation (EC) No. 1924/2006, Regulation (EC) No. 1925/2006, Directive 2002/46/EC, and Regulation (EC) No. 258/97. Regulation (EU) No. 609/2013, proposed by the European Commission, came into force on 20 July 2016. In spite of the existence of this legal framework, the regulation lacks a normative sector regarding the use and application of nutritional ergogenic aids by sports food consumers. These legislative documents should also take into account the WADA considerations on the quality control programs of these substances.

The direct marketing and free sale of sports food could be a health risk in the case of indiscriminate consumption by athletes. So, Regulation (EC) No. 1924/2006, Regulation (EU) No. 1169/2011, and the guidance on the scientific requirements for health claims related to physical performance are essential for the commercialization and advertising of sports food. It is essential to prevent the introduction or dissemination of false, ambiguous, or inexact information and contents that induce an error in the receivers of the information. 

We wish to highlight the importance of the steps taken by the authorities and institutions regarding legislative measures for sports food products. Among them stand out the institutionalization of the EFSA as a European body and the work developed by international scientific societies/companies such as the WADA. All of them have contributed information about consumer guidelines, prescribing practices, prudent use recommendations, and the advantages and limitations of nutritional ergogenic aids. 

However, the results obtained show the absence in the European legislation of a normative sector applied directly to nutritional ergogenic aids for sportspeople. To establish a policy recommendation and to move this process forward, an appropriate institutional setting is needed. Consumer protection provisions should promote greater levels of policy development, regulatory enforcement, and consumer education. Public health measures must be based on the principles of precaution, evaluation, transparency, and the safety of nutritional ergogenic supplements.

## Figures and Tables

**Table 1:** Legislative documents on advertising.

Legislative Documents	Information Related to Nutritional Ergogenic Aids
Law 34/1988, of 11 November of General Advertising. Official State Bulletin No. 274. [20]	Applicable law in Spain about advertising. It establishes the requirement about legal and illegal advertising (misleading, unfair, subliminal) in product labeling. Article 8 speaks about the need to declare derived risks arising out of their normal use.
Law 3/1991, of 10 January of Unfair Competition, Official State Bulletin No. 10. [21]	Unfair Commercial Practices Spanish Directive. This Directive states the laws on unfair commercial practices, including unfair advertising, which directly harm the consumers‘ economic interests and thereby indirectly harm the economic interests of legitimate competitors. The law describes the situations that can be considered unfair competition and the actions deriving from it.
Ministry of Health and Consumer Affairs and the Spanish Federation of Food and Drink Industries. Interpretative agreement on the advertising of the properties of food in relation to health. 1998. [22]	Voluntary agreement applicable in Spain on health claims in advertising of food. This labeling legislation prohibits the attribution to any foodstuff of the property of preventing, treating, or curing a human disease or referring to such properties. -Each health claim should be adequately and sufficiently demonstrated, based on and substantiated by generally accepted scientific evidence. -In order to ensure that the claims made are truthful, it is necessary that the substance that is the subject of the claim is present in the final product in quantities that are sufficient, or that the substance is absent or present in suitably quantities, to produce the nutritional or physiological effect claimed. -Health claims in labeling and advertising should be accompanied by a declaration on the importance of a varied and balanced diet to address feeding requirements. -A particular brand should not be suggested if similar products can produce the same health effects.
Royal Legislative Decree 1/2007 of 16 November, approving the revised text of the General Law for the Protection of Consumers and Users and other complementary laws. Official State Bulletin No. 287. [23]	Law applicable in Spain about relations between users or consumers and businesspeople. The law establishes the basic rights of consumers and the protection against risks that may affect their health or safety.
Law 7/2010, of 31 March, General Audiovisual Communication. Official State Bulletin No. 79. [24]	Law regulating the audio-visual communication of state coverage. It establishes the basic rules in the audiovisual field. Article 18 talks about commercial practices that are prohibited, such as “Any commercial practice that encourages behavior prejudicial to health or safety is prohibited”.
Royal Decree 1907/1996, of 2 August, on advertising and sales promotion products, activities or services intended for health purposes. Official State Bulletin No. 189. [25]	Article 4. Prohibitions and Limitations of advertising intended for health purposes. Any advertising, direct or indirect, massive or individualized promotion of products or substances that suggest/indicate their use/consumption improves physical, mental, or sexual performance sports is prohibited.

**Table 2:** Legislative documents on composition, labeling, and advertising of foods.

Legislative Documents	Information Related to Nutritional Ergogenic Aids
Commission of the European Communities. White Paper on food safety of 12/1/2000. [26]	The establishment of an independent European Food Authority is considered by the Commission to be the most appropriate response to the need to guarantee a high level of food safety. To outline a comprehensive range of actions needed to complement and modernize existing EU food legislation, to make it more coherent, understandable and flexible, to promote better enforcement of that legislation, and to provide greater transparency to consumers; in addition, to guarantee a high level of food safety.
Regulation (EC) No. 178/2002 of the European Parliament and of the Council of 28 January 2002 laying down the general principles and requirements of food law, establishing the European Food Safety Authority and laying down procedures in matters of food safety. [27]	Establishing the European Food Safety Authority and laying down procedures in matters of food safety. This Regulation provides the basis for the assurance of a high level of protection of human health and consumers’ interest in relation to food, taking into account in particular the diversity in the supply of food including traditional products, whilst ensuring the effective functioning of the internal market. Note the creation of a Scientific Committee and Scientific Panels as the Panel on dietetic products, nutrition and allergies (Section 2).
Regulation (EC) No. 1924/2006 of the European Parliament and of the Council of 20 December 2006 on nutrition and health claims made on foods. [28]	This Regulation harmonizes the provisions laid down by law, regulation or administrative action in Member States that relate to nutrition and health claims in order to ensure the effective functioning of the internal market whilst providing a high level of consumer protection. Also apply to nutrition and health claims made in commercial communications, whether in the labeling, presentation or advertising of foods to be delivered as such to the final consumer, including foods that are placed on the market unpacked or supplied in bulk.
Commission Regulation (EC) No. 353/2008 of 18 April 2008 establishing implementing rules for applications for authorization of health claims as provided for in Article 15 of Regulation (EC) No. 1924/2006 of the European Parliament and of the Council. [29]	This Regulation establishes implementing rules for the following: 1. Applications for authorization, submitted in accordance with Article 15 of Regulation (EC) No. 1924/2006; and 2. Applications for the inclusion of a claim in the list provided for in Article 13(3) submitted in accordance with Article 18 of Regulation (EC) No. 1924/2006.
Regulation (EU) No. 1169/2011 of the European Parliament and of the Council of 25 October 2011 on the provision of food information to consumers, amending Regulations (EC) No. 1924/2006 and (EC) No. 1925/2006 of the European Parliament and of the Council, and repealing Commission Directive 87/250/EEC, Council Directive 90/496/EEC, Commission Directive 1999/10/EC, Directive 2000/13/EC of the European Parliament and of the Council, Commission Directives 2002/67/EC and 2008/5/EC and Commission Regulation (EC) No. 608/2004. [30]	This Regulation provides the basis for the assurance of a high level of consumer protection in relation to food information, taking into account the differences in the perception of consumers and their information needs. Establishes the general principles, requirements and responsibilities governing food information, and in particular food labeling. It lays down the means to guarantee the right of consumers to information and procedures for the provision of food information, taking into account the need to provide sufficient flexibility to respond to future developments and new information requirements (Chapter IV, Sections 1, 2 and Chapter VII).
EU Register on nutrition and health claims. [31]	European Union Register of nutrition and health claims made on foods, showing: 1. Permitted nutrition claims and their conditions of use; 2. Authorized health claims, their conditions of use and applicable restrictions, if any; 3. Non-authorized health claims and the reasons for their non-authorization; 4. EU legal acts for the specific health claims; and 5. National measures mentioned in Art. 23(3) of Regulation EC 1924/2006. URL: http://ec.europa.eu/nuhclaims/

**Table 3:** Specific legislative documents on nutritional ergogenic aids.

Legislative Documents	Information Related to Nutritional Ergogenic Aids
Royal Decree 2685/1976 Technical health regulations on foodstuffs intended for particular nutritional uses. Official State Bulletin No. 284. [32]	This Regulation defines food prepared for dietary regimens and/or special uses and establishes the legal regulation of such products.
Directive 2009/39/EC of the European Parliament and of the Council of 6 May 2009 on foodstuffs intended for particular nutritional uses. [33]	The Directive defines foodstuffs for particular nutritional uses are foodstuffs. Annex 1. Groups of foodstuffs for particular nutritional uses for which specific provisions will be laid down by specific Directives: Group 5 “foods intended to meet the expenditure of intense muscular effort, especially for sportsmen”.
Commission Regulation (EC) No. 953/2009 of 13 October 2009 on substances that may be added for specific nutritional purposes in foods for particular nutritional uses. [34]	Article 1. Regulation shall apply to foods for particular nutritional uses, excluding those covered by Directive 2006/125/EC and Directive 2006/141/EC. Article 2. Among the substances belonging to the categories appearing in Annex to this Regulation, only those listed in that Annex, complying with the relevant specifications as necessary may be added for specific nutritional purposes in the manufacture of foodstuffs for particular nutritional uses covered by Directive 2009/39/EC. Without prejudice to Regulation (EC) No. 258/97 of the European Parliament and of the Council (6), also substances not belonging to the categories appearing in the Annex to this Regulation may be added for specific nutritional purposes in the manufacture of foods for particular nutritional uses.
Regulation (EU) No. 609/2013 of the European Parliament and of the Council of 12 June 2013 on food intended for infants and young children, food for special medical purposes, and total diet replacement for weight control and repealing Council Directive 92/52/EEC, Commission Directives 96/8/EC, 1999/21/EC, 2006/125/EC and 2006/141/EC, Directive 2009/39/EC of the European Parliament and of the Council and Commission Regulations (EC) No. 41/2009 and (EC) No. 953/2009. [35]	This regulation excludes products for athletes from the category of “dietetics”. Applicable from 20 July 2016. With regard to food intended to meet the expenditure of intense muscular effort, especially for sportsmen, no successful conclusion could be reached as regards the development of specific provisions due to widely diverging views among the Member States and stakeholders concerning the scope of specific legislation, the number of subcategories of food to be included, the criteria for establishing compositional requirements and the potential impact on innovation in product development. Therefore, specific provisions should not be developed at this stage. Meanwhile, on the basis of requests submitted by food business operators, relevant claims have been considered for authorization in accordance with Regulation (EC) No. 1924/2006. Different views exist as to whether additional rules are needed to ensure an adequate protection of consumers of food intended for sportsmen, also called food intended to meet the expenditure of intense muscular effort. The Commission should, therefore, be invited, after consulting the Authority, to submit to the European Parliament and to the Council a report on the necessity, if any, of provisions concerning food intended for sportsmen. The consultation of the Authority should take into account the report of 28 February 2001 of the Scientific Committee on Food on composition and specification of food intended to meet the expenditure of intense muscular effort, especially for sportsmen. In its report, the Commission should, in particular, evaluate whether provisions are necessary to ensure the protection of consumers. Taking into account the existing situation on the market and Directives 2006/125/EC and 2006/141/EC, and Regulation (EC) No. 953/2009, it is appropriate to establish and include in the Annex to this Regulation a Union list of substances belonging to the following categories of substances: vitamins, minerals, amino acids, carnitine and taurine, nucleotides, choline and inositol. Article 13. Food intended for sportspeople. By 20 July 2015, the Commission shall, after consulting the Authority, present to the European Parliament and to the Council a report on the necessity, if any, of provisions for food intended for sportspeople. Such a report may, if necessary, be accompanied by an appropriate legislative proposal.
EFSA Panel on Dietetic Products, Nutrition and Allergies (NDA). Guidance on the scientific requirements for health claims related to physical performance. EFSA Journal 2012;10(7):2817. [36]	Document prepared by The Panel on Dietetic Products, Nutrition and Allergies (NDA) of the EFSA about scientific requirements for health claims related to physical performance.
Scientific and technical assistance on food intended for sportspeople. Question Number: EFSA-Q-2015-00403. [37]	Technical report of the EFSA to compile existing scientific advice in the area of nutrition and health claims and Dietary Reference Values for adults that is relevant to sportspeople and to inform the Commission on how such scientific advice relates to the different conclusions and specifications of the report of the Scientific Committee on Food (SCF) of 2001 on the composition and specification of food intended to meet the expenditure of intense muscular effort, especially for sportspeople.
